# The role of insurance in the achievement of universal coverage within a developing country context: South Africa as a case study

**DOI:** 10.1186/1471-2458-12-S1-S5

**Published:** 2012-06-22

**Authors:** Alex M van den Heever

**Affiliations:** 1 Old Mutual Chair of Social Security Policy Management and Administration, Graduate School of Public and development Management, University of the Witwatersrand, Johannesburg, South Africa

## Abstract

**Background:**

Achieving universal coverage as an objective needs to confront the reality of multiple mechanisms, with healthcare financing and provision occurring in both public and private settings. South Africa has both large and mature public and private health systems offering useful insights into how they can be effectively harmonized to optimise coverage. Private healthcare in South Africa has also gone through many phases and regulatory regimes which, through careful review, can help identify potential policy frameworks that can optimise their ability to deepen coverage in a manner that complements the basic coverage of public arrangements.

**Research question:**

Using South Africa as a case study, this review examines whether private health systems are susceptible to regulation and therefore able to support an extension and deepening of coverage when complementing a pre-existing publicly funded and delivered health system?

**Methods:**

The approach involves a review of different stages in the development of the South African private health system and its response to policy changes. The focus is on the time-bound characteristics of the health system and associated policy responses and opportunities. A distinction is consequently made between the early, largely unregulated, phases of development and more mature phases with alternative regulatory regimes.

**Results:**

The private health system in South Africa has played an important supplementary role in achieving universal coverage throughout its history, but more especially in the post-Apartheid period. However, the quality of this role has been erratic, influenced predominantly by policy vacillation.

The private system expanded rapidly during the 1980s mainly due to the pre-existence of a mature health insurance system and a weakening public hospital system which could accommodate and facilitate an increased demand for private hospital services. This growth served to expand commercial interest in health insurance, in the form of regulated medical schemes, which until this point took the form of non-commercial occupational (employer-based) schemes. During the 1980s government acquiesced to industry lobbies arguing for the deregulation of health insurance from 1989, with an extreme deregulation occurring in 1994, evidently in anticipation of the change of government associated with the democratic dispensation. Dramatic unintended consequences followed, with substantial increases in provider and funder costs coinciding with uncontrolled discrimination against poor health risks.

Against significant industry opposition, including legal challenges, partial re-regulation took effect from 2000 which removed the discretion of schemes to discriminate against poor health risks. This included: the implementation of a strong regulator of health insurance; the establishment of one allowable vehicle able to provide health insurance; open enrolment, whereby schemes could not refuse membership applications; mandatory minimum benefit requirements; and a prohibition on setting contributions or premiums on the basis of health status. After a two-year lag, dramatically reduced cost trends and contributions became evident. Aside from generally tighter regulation across a range of fronts, this appears related to the need for schemes to compete more on the basis of healthcare provider costs than demographic risk profiles. Despite an incomplete reform improved equitable coverage and cost-containment was nevertheless achieved.

A more complete regulatory regime is consequently likely to deepen coverage by: further stabilising and even decreasing costs; enhanced risk pooling; and access for low income groups. This would occur if South Africa: improved the quality of free public services, thereby creating competitive constraints for medical schemes; introduced risk-equalisation, increasing the pressure on schemes to compete on the cost and quality of coverage rather than their risk profile; and through the establishment of improved price regulation.

**Conclusions:**

The objective of universal coverage can be seen in two dimensions, horizontal extension and vertical deepening. Private systems play an important role in deepening coverage by mobilising revenue from income earners for health services over-and-above the horizontal extension role of public systems and related subsidies. South Africa provides an example of how this natural deepening occurs whether regulated or unregulated. It also demonstrates how poor regulation of mature private systems can severely undermine this role and diminish achievements below attainable levels of social protection. The mature South African system has demonstrated its sensitivity to regulatory design and responds rapidly to changes both positive and negative. When measures to enhance risk pooling are introduced, coverage is expanded and becomes increasingly fair and sustainable. When removed, however, the system becomes less stable and fair as costs rise and people with poor health status are systematically excluded from cover. This susceptibility to regulation therefore presents an opportunity to policymakers to achieve social protection objectives through the strategic management of markets rather than exclusively through less responsive systems based on tax-funded direct provision. This is especially relevant as private markets for healthcare are inevitable, with policy discretion reduced to a choice between functional or dysfunctional regimes.

## Definitions and terms used

Administrators: Refers to third-party administrators who contract with medical schemes to perform all major operational functions. This includes member management, claims processing, and provider negotiations.

Medical schemes: The regulated health insurance vehicle responsible for providing health insurance in South Africa.

Non-price competition: Refers to hospital competition with other hospitals for specialists to achieve patient demand targets. This is to be distinguished from price competition whereby hospital would compete with other hospitals for patient volumes by offering volume-related rice discounts.

Occupational scheme: Refers to an employer-sponsored medical scheme which by law is now a “restricted membership scheme”. Historically these were the first coherent health insurance arrangements in South Africa.

Open scheme: A type of medical scheme required by law, from 2000, to accept any applicant for enrolment as a member.

Restricted membership scheme: A type of medical scheme able by law to limit membership to a specified group associated with a specific employer, industry, profession or trade union. A scheme can only be permitted to restrict membership where it will enhance risk pooling and social solidarity.

Risk equalisation fund: A statutory mechanism proposed but never implemented in South Africa to transfer funds from schemes with a risk profile below the market average to schemes with a risk profile above the market average in order to achieve system-wide risk pooling across multiple funds.

Scale of benefits: The tariff schedule used by medical schemes to reimburse hospitals and health professionals. Prior to 1994 this was statutory with providers charging according to the schedule. From 1994 this became a reference price schedule with both schemes and providers able to set their own reimbursement levels and tariffs.

## Background

South Africa is an upper-middle-income country with a population of roughly 50.6 million (2011 mid-year estimate) [1] and a per capita Gross Domestic Product (GDP) of US$7,612 (2010). Overall population growth is 1.10% (2010-2011) which has declined from 1.33% (2001-2002) [2]. The urban population reached 57% in 2001 and is increasing steadily [2]. It is probable that the 2011 census will reveal a level well in excess of 60%.

Due largely to high structural unemployment rates, which for a narrow definition of unemployment stood at 25% in the 3^rd^ quarter of 2011 [3], income inequality is high with an estimated gini coefficient for 2008 of 0.67-0.68 [4]. This is to some degree mitigated by an extensive system of social assistance grants reaching upward of 15 million people by 2011, with expenditure equivalent to 3.6% of Gross Domestic Product (GDP) [5].

South Africa’s burden of disease has deteriorated since 1994 due largely to HIV and AIDS and associated co-morbidities such as tuberculosis (TB). In 2011 HIV and AIDS prevalence is estimated at 10.6%, with life expectancy for males and females at 54.9 and 59.1 respectively [1]. However, maternal mortality ratios (MMRs) well below expectations for country of South Africa’s level of development, estimated at 300 out of 100,000 live births with a confidence interval of 15 to 500 [6], suggest that public sector clinical services are underperforming at existing levels of investment [7]. Although 43.7% of all maternal deaths involve AIDS, patient-related avoidable factors were recorded in 46.5% of maternal deaths with 38.4% clearly avoidable [8]. Human Rights Watch investigations have also revealed that pregnant women are abused by nurses in public facilities with inadequate formal accountability mechanisms to protect patients [9].

**Figure 1:**
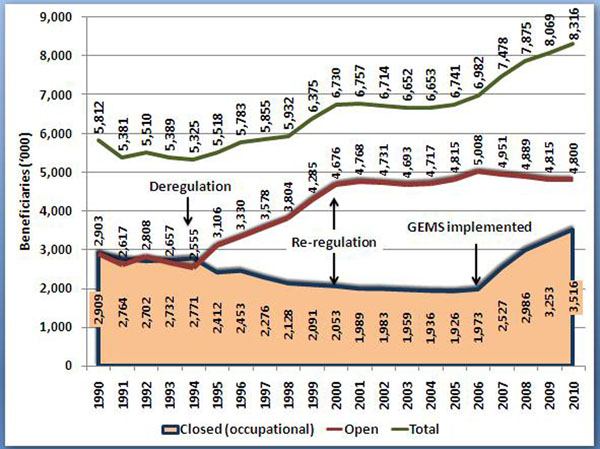
**Medical scheme beneficiaries from 1990 to 2010** Data from Council for Medical Schemes’ Annual Reports [29]. The deregulation of 1994 resulted in the movement of beneficiaries from closed schemes to open schemes. The drop in beneficiary growth from 2000 to 2006 resulted from a stagnation of economic growth. From 2006 most beneficiary growth results from the introduction of the Government Employee’s Medical Scheme (GEMS), with some growth also resulting from increased economic growth. The introduction of GEMS, which is a closed scheme, also resulted in a switch away from open commercial schemes.

South Africa has large public and private health systems, with the former funded by general taxes for nine provincial health departments and the national department, with spending equivalent to 3.8% of GDP in 2010 [10], and the latter through regulated voluntary not-for-profit private health insurance arrangements, referred to as medical schemes, equivalent to 3.4% of GDP in 2010 [11]. In 2010 there were 8.3 million medical scheme beneficiaries equivalent to 16.6% of the total population. This number is also growing continuously (figure 1). Overall health expenditure is consequently around 8.8% of GDP including out-of-pocket (OOP) expenditure estimated at 1.5% of GDP in 2009 [12].

Out-of-pocket expenditure, which is well within international norms, predominantly occurs in the top income decile, although spending on private general practitioners and specialists occurs across all income groups despite access to free public services [13]. As public hospital services are free for low-income groups while medical schemes prioritise insurance for catastrophic expenses, most OOP expenditure is for basic doctor-based primary care services and some medicines.

Given this configuration of protection, South Africa technically complies with the goal of universal coverage as a comprehensive package of health services is available on a pre-paid basis either through the public sector or regulated health insurance (medical schemes). However, poor policy development and implementation in the post-apartheid period (from 1994) have exacerbated rather than addressed historical institutional weaknesses [7,9,30].Government critics argue that inadequate attention has been given to the correct mix of institutional mechanisms to permit both the public and private systems to realise their full potential. Whereas much can be said about the public system, this article focuses exclusively on the potential role of the private system and its contribution to extending and deepening universal coverage.

Whereas the role of public subsidies, whether to fund in-kind health services or to finance private provision, is to maximise the horizontal extension of health services to those without adequate income, privately funded healthcare complements this role through the mobilisation of additional revenue. This funding can extend the reach of government-subsidised services where income earners use private services, or fund supplementary provision where coverage is highly rationed. South Africa’s experience is more consistent with the former. However, full advantage may not have been taken of the opportunities presented over time. This review therefore makes use of an historical analysis of the South African private health system and its regulation to identify whether private markets are susceptible to intervention such that coverage is extended and deepened in a sustainable manner.

## Research question

Using South Africa as a case study, this review examines whether private health systems are susceptible to regulation and therefore able to support an extension and deepening of coverage when complementing a pre-existing publicly funded and delivered health system?

## Methods

The approach involves a review of different stages in the development of the South African private health system and its response to policy changes. The focus is on the time-bound characteristics of the health system and associated policy responses and opportunities.

The review is divided broadly into two periods:

• From 1889 to 1999: where the system moves from an immature non-commercial regime into a highly commercialised insurance and provision in the final ten years and where the latter period is heavily affected by a major deregulation of health insurance; and

• From 2000 to 2010: where the mature system is subjected to new regulatory interventions.

The periods and regulatory regimes are evaluated qualitatively with the relevant drivers of change identified, focusing on their susceptibility to regulatory interventions.

## The history of medical schemes to 1999

### The emergence of occupational and industry schemes

What are today regulated in law as medical schemes had their origins in various forms of occupational health insurance beginning as early as 1889 with the De Beers Consolidated Mines Limited Benefit Society Benefit Society within the territory of what is now South Africa. Within fifteen years seven more funds of this nature were established as the mining economy grew. Health insurance arrangements preceded the establishment of any government structure to supervise health care which occurred for the first time only in 1919 following the establishment of the Union of South Africa in 1911, which consolidated four separate countries following the Anglo Boer war, and the influenza epidemic of 1919. By 1940 around forty eight occupational schemes existed. Only from 1956 were medical schemes regulated for the first time. Due to the rapid growth of South Africa’s economy the number of schemes increased to 169 by 1960 with beneficiaries almost exclusively white. By 1990 the number of schemes peaked at around 230 with some schemes serving more than one employer [15].

The first coherent regulatory framework was implemented in 1967 which consolidated the legal definitions, historically spread through various pieces of legislation, and established registration and compliance structures. During this period and up until around 1984 medical schemes were non-competing occupational funds sponsored by employers or industries. They were supervised as trust arrangements with boards representing the interests of beneficiaries and employers. No profits were permitted or surpluses distributed. Benefits and provider tariffs were also regulated by way of a highly contested scale of benefits prepared by the Representative Association of Medical Schemes (RAMS) a private association representing all medical schemes. Tension between RAMS and healthcare providers resulted in ongoing government vacillation between various models of collectivised tariff setting [15] .

As medical schemes were predominantly non-competing at this time, with risk pools framed around medium-to-large employers and industries, benefit designs and the setting of contributions complied largely with the principals of social solidarity. Contributions were differentiated on the basis of income, with higher income groups paying more. Differentiation on the basis of health status was prohibited by law. Schemes also needed to comply with a system of mandatory minimum benefits based on the scale of benefits, which specified the proportion of reimbursement required of a consultation, procedure, or tariff. From 1980 schemes were required to pay in full any invoice submitted directly by registered medical practitioners compliant with the scale of benefits. Where an invoice exceeded the scale of benefits schemes reimbursed the member only after they had initially settled the account. This mechanism served as an incentive for healthcare service providers to comply with the scale of benefits as members could be slow and irregular in settling accounts [15].

Over this period medical schemes primarily reimbursed the expenses of private health professionals and hospital services located in the public sector. Access to public hospitals was, and still is, subject to a means test affecting everyone over the tax threshold and quite a few below [15]. For anyone over the tax threshold a medical scheme was therefore essential to avoid catastrophic health expenses associated with private specialist and public hospital services.

To encourage employers to provide medical scheme coverage a tax subsidy was available where an employer paid the medical scheme contribution. This has since been altered to an allowable deduction in the hands of an employee to cater for the self-employed. All low-income groups have always had access to a free public health service which, apart from a few exceptions, was formally segregated on the basis of race from 1948. Interestingly, due to this configuration white taxpayers did not have free access to public services, although they did have a portion of their contribution indirectly subsidised through the tax rebate.

### The emergence of private hospitals

International sanctions, together with the financing of wars in Angola and Mozambique, reduced South Africa’s economic growth rate from 1982 with a negative impact on government finances. Roughly from that period the tax-based funding of public health services began to worsen. This coincided with the emergence of private hospitals owned largely by medical specialists already working in the public sector. Many specialists working part-time in the private sector during the 1980s were also in a position to divert medical scheme covered patients into their private practices and associated private hospital services. By the end of the 1980s the decline in the quality of public hospitals together with the financial incentives of private specialists had helped the rapid creation of a fairly substantial private doctor-owned hospital system.

From 1986 to 1989 private hospitals increased from 65 (6,125 beds or 5% of the total) to 101 (10,908 beds or 9% of the total). By 2010 private hospitals had increased to 216 with 31,067 beds or 26% of the total. The number of public hospital beds have however always far exceeded the private sector with 117 842 (91% of all beds) available in 1986. They have however declined to 88 920 beds (or 74% of the total) by 2010. The decline in the number of public sector beds was consequently matched by increased beds in the private sector (Table 1).

**Table 1:** Private and public hospitals and bed estimates from 1976 to 2010

Year	Private		Public	
	Hospitals	Beds	Hospitals	Beds
**1976**	25^1^	2 346^1^		
**1986**	65^1^	6 125^2^		117 842^3^
**1989**	101^1^	10 936^1^		
**1998^4^**	162	20 908	343	107 634
**2010^5^**	216	31 067	410	88 920

^1^ Hospital Association of South Africa [32], ^2^ Estimated using the bed to hospital ratio for 1976 from the other data in the table, ^3^Estimated from Zwaranstein et al [33], ^4^Health Systems Trust [34], ^5^Health Systems Trust [35].

The shift of treatment into private hospitals dramatically increased the cost of medical schemes as public hospitals did not charge full cost recovery (implicitly subsidising medical scheme members) and had no incentives to over-service. Private hospitals however priced for both cost-recovery and profit, and benefited from the incentives of private specialists to over-service. The resulting cost changes during the 1980s began to alter the market dynamics of medical schemes. They also increased the turnover and complexity of funders, incentivising increased commercial interest in the provision of health insurance.

### The emergence of commercial competing open schemes

During the 1980s medical scheme administrators, who contracted with occupational medical schemes to manage their operations, developed sufficient expertise in health insurance to expand into commercial insurance models independent of employers. Coverage was offered to multiple employers reflecting a natural evolution of the system. However, they were constrained by the occupational-scheme orientation of the legislative framework. Commercial schemes consequently lobbied for the ability to design contribution tables for different employers and even individuals, while keeping benefit arrangements common. For this to happen the strict legislative regime, which limited the manner in which contributions could be set, came under pressure from the commercial arm of medical schemes, the third-party administrators contracted to schemes to perform their operational functions. Proposals to liberalise the health insurance market surfaced from 1984, couched in the pro-market language prevalent at the time, which argued that this would make medical schemes more affordable. The objectives underpinning the amendments to the Medical Schemes Act of 1967 stressed these “positive” intentions: “To have a medical scheme the ordinary person will be able to afford”; and “To prevent the socialisation of medicine” [15]. This led to the first significant deregulation of health insurance in South Africa.

### Medical scheme deregulation – the period from 1989 to 1999

In accepting the “pro-market” arguments, the ability to set premiums on the basis of the risk of claiming were implemented by government in 1989. From this period contributions could be set on the grounds of the number of dependents, income, age, geographic area, claims experience, extent of cover provided, period of membership, and the size of the participating group [15].

This remarkable deregulation however was not sufficient to permit commercial or open medical schemes (which serve multiple employers and sometimes individuals) to emerge. The requirement to comply with mandatory minimum benefits determined in relation to the scale of benefits constrained their ability to undercut the occupational schemes. Risk rating was not enough.

Industry lobbies successfully influenced the government to implement a material further deregulation from 1 January 1994, just prior to the new democratic dispensation that would bring in a new government from May 1994. This removed the requirement for mandatory minimum benefits [15]. In 1993 government also made membership of a medical scheme voluntary for government employees, allowing them to choose their own open scheme. The former mandatory government-sponsored schemes, of which there were four, were consequently converted into open commercial schemes. Three other public sector medical schemes remained closed and mandatory: the police force scheme; the parliamentary scheme which includes all judges; and the scheme for correctional services (prisons) staff.

The changes rapidly transformed the system of medical schemes with inter alia.: a substantial shift away from occupational schemes into open commercial schemes (from 50% of all beneficiaries in 1994 to over 70% by 1999) (figure 1); the use of illegal commissions to incentivise employers to close their occupational schemes and shift to open schemes [16]; and the explicit discrimination against older and sicker members within open competing medical schemes [15].

Price competition between commercial medical schemes occurred on the basis of risk-selection (the selection of beneficiaries on the basis of health status) and risk-rating (the pricing of contributions on the basis of health status). Contributions paid through open schemes were flat-rate (apart from variations due to health status) and eliminated the income cross-subsidies that characterised occupational schemes. Social solidarity principles inherent within medical schemes were consequently wiped out in a mere five years.

Although medical schemes could not lawfully distribute profits, open commercial medical schemes, dominated by their administration companies, began introducing reinsurance contracts to siphon off underwriting surpluses into entities, that colluded with administrators or were owned by them, and to pay brokers [17]. Another mechanism to circumvent the laws prohibiting the distribution of profits and the funding of brokers involved the commission-based co-selling of short- and long-term (for profit) health insurance products packaged together with medical schemes in collusion with the administrators. This intensified the commercial incentives to discriminate against people and groups with a higher risk of claiming benefits.

### Risk-based scheme competition and costs

**Figure 2:**
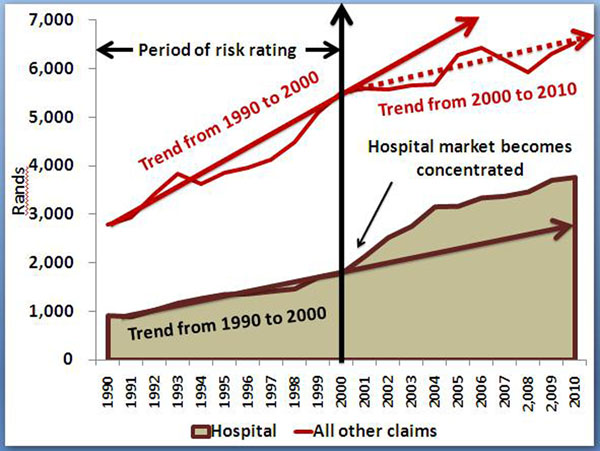
**Private hospital and other medical schemes claims costs per person per annum, South African Rands (2010 prices)** Data from Council for Medical Schemes’ Annual Reports [29]. Medical scheme claims costs increased more steeply during the 1990s than during the post-reform period which was implemented from 2000. Private hospital costs initially increased more steeply during the post reform phase after the market became concentrated from 2000. Private hospitals returned to the pre-2000 trend in 2004 but at a structurally higher cost.

Contradicting the apparent rationale for deregulation, that price competition through risk rating (and selection) will bring down prices and increase membership, the opposite occurred. The period from 1994 saw a trend break in medical scheme per capita contribution increases (figure 2). Non-health care expenditure (administration expenses and net reinsurance losses) by medical schemes also demonstrate a break in trend from 1994 [15]. Importantly, new expenses over-and-above basic administration suddenly made their appearance from 1994.

Over this period regulation involved little more than the registration of medical schemes and some low-key prudential supervision. The regulator itself was a low-level official in the National Department of Health with a staff of seven, none of whom were professionally qualified [18]. Although a Council existed to supervise the regulator, the majority of appointments were of individuals working directly within medical schemes or medical scheme administrators. It could be argued that the market was essentially self-regulated at this stage.

Dramatic contribution increases from 1994 to 1999 consequently resulted from two sources: trend changes in the cost of healthcare services; and trend changes in non-health costs. Whereas the latter is explained by the latitude given to medical schemes and their administrators to profit from schemes, the former was driven largely by the nature of scheme price competition. As schemes could compete by passing risk onto members and beneficiaries (through risk-selection and risk-rating) they had no incentive to control underlying medical expenses. The inelastic nature of demand for medical scheme coverage furthermore meant that cost increases did not result in membership declines. Competing medical schemes therefore faced no market-related penalties for passing cost increases on to contributors, provided they kept their increases in line with those of other schemes. The pricing strategies therefore focused exclusively on gaining market share from occupational schemes rather than competition amongst commercial schemes, further dulling incentives to be cost-efficient.

The incentive-driven behavioural change of schemes affected the behaviour of medical service providers during this period as schemes had little interest in serious cost containment. Poor regulation of specialists from a competition perspective allowed successful horizontal collusion (between specialists) to foreclose early attempts by schemes in 1997 to introduce forms of selective contracting [19]. Such scheme initiatives could have influenced subsequent hospital and specialist cost trends.

**Figure 3:**
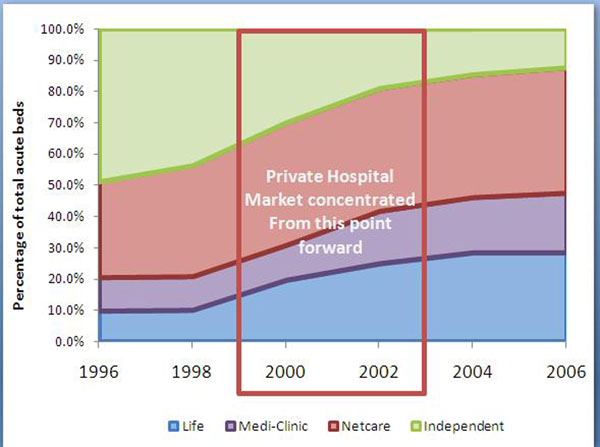
**Private hospital changes in market share from 1996 to 2006 for the three main hospital groups: Life Health, Netcare, and Medi-clinic** Data from Council for Medical Schemes [16] From 2000 the market for private hospital acute beds was concentrated in the hands of only three hospital groups.

Whereas doctor-owned hospitals emerged during the 1980s, corporate ownership of hospitals became a major trend only during the 1990s, with significant and rapid market consolidation into three corporate groups by 1999, a period of roughly ten years. Although the hospital market was technically not an oligopoly in 1999, it was a mere two years thereafter [16]. From 1994 only three hospital groups bought independent hospitals. At some point during the early 1990s a market power threshold was crossed which accelerated the consolidation by the three groups. (Figures 2 and 3).

This market power was initially directed at independent hospitals who were vulnerable to non-price competition against the more revenue secure hospital groups. Their increasing and stable surpluses were used to chase demand by competing for specialists rather than competing for patient volumes (demand) though price discounts. Specialists, who operate independently of hospitals in the private sector (they are not employed by hospitals), are actively attracted to hospital groups by capital investments in specialised medical equipment and practice management support. As certain hospital groups achieved sustainable revenue flows through the predictable demand provided by the specialist referrals, they were better positioned than independent hospitals to invest in the capital equipment required to attract more specialists. As independent hospitals lost their ability to retain specialists, demand for their services declined, exposing them to inevitable takeover by any one of the cash flush large hospital groups. As a result whereas in 1998 44.7% of private hospital beds were still in independent hands, by 2002 only 30.2% remained. By 2006 this had dropped to 16.2% (figure 3) [16].

Once the hospital consolidation passed an important concentration threshold in 1999, their market power, initially directed against independent hospitals (competitors), could now be directed against medical schemes who became price takers. Thus although a trend break in rising private hospital costs occurs only from 2000, the conditions for this trend break were established during the 1990s. (Figures 2 and 3).

### Scheme demographics

The demographic make-up of medical schemes also became more inclusive from the mid-1980s to 1999 with a surging black middle class taking up coverage. Whereas in 1984 only 14.8% of scheme beneficiaries were black [20], by 1995 it improved significantly to 37.6% with whites at 48.6% [21]. These positive trends continued through 2000, with black beneficiaries substantially exceeding whites by 2007 with proportions at 42.8% and 39.8% respectively. By 2010 black beneficiaries constituted nearly half of all beneficiaries at 46.3% (3.8 million beneficiaries) with whites now well below at 35.7% (3 million beneficiaries) [22]. The differential improvement in black membership from 1995 is attributable to growing black middle class. It is likely that by 2012 black beneficiaries will exceed 50% of the total.

## Medical scheme re-regulation – the period from 2000

### Policy positions

During the 1990s plans were made to reintroduce a regulatory regime framed around a preferred private health insurance entity that had evolved to date, the medical scheme. Parallel insurance operating through more conventional insurance regimes were regarded as unregulatable as they had no standard institutional structure or regulatory regime. Short- and long-term insurance arrangements were also subject to much lighter regulatory supervision through the Financial Service Board reporting to the Minister of Finance.

However, a return to the pre-1994 framework could no longer accommodate the altered and rapidly evolving nature of the private health system. The existence of competing commercial medical schemes, together with a supportive broker system, was now an unavoidable reality. The continuous decline in occupational schemes, particularly the smaller ones, was now systemic. Cost containment had also become an evident problem, with non-health costs also reaching alarming levels [16].

The new regulatory model now needed to reconstitute social solidarity that also generated incentives to manage costs. A policy framework tabled in 1995 talked to new measures (that had never existed before) as well as some that had been removed in 1989 and 1994 including: community rating (contributions that could not be differentiated on the basis of health status); open enrolment, whereby open commercial medical schemes would not be able to deny membership to applicants (removing their ability to risk-select); mandatory minimum benefits with an emphasis on catastrophic health expenses; inter-scheme risk-equalisation, which sought to affect inter-scheme financial transfers to ensure that all schemes faced the same prospective demographic risk profile; and mandatory membership to deal with anti-selection (where people only take up cover when needing health care). The proposed framework also incorporated recommendations for income-based cross-subsidies, to be achieved through a risk-equalisation mechanism, and mandatory participation for income earners [23].

### What was eventually implemented?

In the face of significant industry opposition, which included a failed legal challenge to stop the legislation, a less comprehensive framework than originally proposed was eventually legislated in 1998, taking effect from 2000. Important provisions included the establishment of an independent regulator separate from government; open enrolment; community rating; minimum solvency requirements; the accreditation of brokers and the regulation of their commissions; and mandatory minimum benefits focusing on catastrophic medical expenses. However, the framework did not mandate membership and nor did it introduce risk-equalisation. To mitigate the inevitable anti-selection within a voluntary environment, a system of waiting periods and late-joiner penalties were introduced. In both instances the penalty framework incorporated rewards for continuity of membership within the system of medical schemes. All significant barriers to free movement between schemes was removed.

The recommendations regarding mandatory membership and risk-equalisation were taken up in a subsequent committee of inquiry which concluded its work in 2002 but was never implemented [24].

### The impact of re-regulation

Even the partial framework, which really started impacting after 2001, systematically altered important trends. From 2002 non-health real per capita expenditure largely flattened with only a 4.6% real increase over the entire period to 2010. Demonstrating the extent of the behavioural change, in the two years leading to 2002 the annual real per capita increases were 28.2% and 21.8% respectively [11].

Contribution increases also flattened from 2002 with a 19.3% real per capita increase over the entire period to 2010 [11]. However, a more definitive trend break occurred after 2004 with the increase over the whole period to 2010 only 4.7%. This can largely be attributed to the flattening of claims cost increases from 2004 (figure 2), and schemes achieving their new statutory solvency levels by 2005 (these had to be phased in from 2001 for a period of five years). Regulatory action to remove all inappropriate reinsurance agreements also took effect from 2004.

The altered year-on-year trends in medical costs, although remaining structurally high, resulted from a number of measures that came to a head during 2004. This included new price legislation for medicines, in place from August 2004, which *inter alia* introduced a single-exit pricing mechanism. This together with other statutory provisions prohibited the “discounting” of medicines, effectively outlawing a number of cost-inducing kick-back arrangements operating between private hospitals and medicine manufacturers as well as doctors and medicine manufacturers [16]. Per capita medicine costs subsequently declined for three consecutive years and remaining largely flat thereafter.

Several measures aimed at medical schemes also altered their incentives and capability to manage costs from 2001. As open commercial schemes could no longer compete using risk-rated contributions or risk-selection, they had to compete on healthcare costs. In the case of medicines the mandatory minimum benefits for chronic conditions, which took effect from 2004, incentivised immediate efficiencies through scheme-developed clinical protocols and medicine formularies.

Generalised improvements in administration from 1999, which saw electronic claims processing introduced universally, also improved the capacity to manage costs. This capability was further enhanced from 2005 with a statutory requirement placed on healthcare providers to supply clinical codes together with invoices.

### Scheme consolidation

The regulatory framework (through new minimum membership requirements), together with commercial incentives already prevalent in the 1990s, saw schemes consolidate, with over 220 schemes in 1998 reducing to only 100 by 2010. The consolidation was also given a boost from 2005 with the introduction of the Government Employees Medical Scheme (GEMS) reversing the 1993 decision liberalising medical cover for government employees with significant implications for the market. As there are approximately 1.3 million general government employees, a maximum potential membership exists, when dependents are included, of around 3 million. GEMS is presently at around half this number and remains the fastest growing scheme in the country.

Roughly coinciding with this period one commercial open scheme, Discovery Health Medical Scheme, also grew rapidly from around 250,000 beneficiaries at the beginning of 2000 to approximately 2.1 million beneficiaries by 2010. By 2010 just two medical schemes therefore covered 41% of all beneficiaries (3.4 million out of 8.3 million). Although the consolidation holds out some possibility of better contracting with providers, it also reduces market penalties for over-priced scheme contributions. The consolidation of membership into a few national schemes reflects an apparent systemic trend resulting from the emergence of multi-employer commercial schemes in the 1980s.

### Differential pricing by group size

Open enrolment, which required that all open schemes accept all applicants, eliminated contribution-based price discrimination based on the size of group joining an open scheme. Prior to 2000 open schemes would, at their discretion, differentiate contributions on the basis of group size, with individuals and smaller groups paying more than larger groups. This practice prejudices small employers and the self-employed. The introduction of open enrolment together with community rated contributions (where the community is a plan or option offered by a scheme) eliminated this differential pricing according to group size without undermining financial sustainability, which has important implications for enhanced social solidarity.

## Gaps in medical scheme regulation and its consequences

### Risk pooling

The legislative framework of 1998 and implemented from 2000 enhanced the social protection possible through a voluntary (predominantly) competing health insurance environment, but retained significant and important gaps.

The removal of discrimination on the basis of health status in the absence of inter-scheme risk-equalisation exposes competing health insurers to their demographic risk profile which they cannot control. Competing health insurers with poor risk profiles consequently need to be priced in excess of those with good risk profiles regardless of how efficiently they manage claims costs. This drives younger and healthier beneficiaries to schemes with better risk profiles, giving rise to a price-related death spiral and scheme failure. Given open enrolment, beneficiaries migrate to surviving schemes resulting in systemic scheme consolidation.

Although unable to explicitly introduce risk-rated contributions, schemes have been able to manage this contingency by offering multiple plans, specified in law as options, with different levels of benefit. The limited range of mandatory minimum benefits (amounting to around 35% of a comprehensive package) allowed schemes to achieve milder forms of risk-rating by leveraging off the anti-selective conduct of younger and healthier families and groups who self-select benefit packages that reflect their risk profile. Options offering only catastrophic health cover therefore predominantly attract younger and healthier groups, while comprehensive benefit options attract older and sicker groups. As each option (or benefit plan) is priced separately, cross-subsidies between options within the same scheme can be set at the discretion of the scheme, allowing contribution rates to vary between options on two factors: the risk of claiming; and benefit levels. Despite community rating, therefore, comprehensive options implicitly include a risk-related loading, while catastrophic cover options benefit from a risk-related discount.

The risk-sifting options designs remove scheme incentives to aggressively risk-select in exchange for an implicit contribution penalty for those choosing comprehensive options. However, the penalty is more subdued than occurred during the deregulations of 1989 and 1994 for a number of market-related reasons. Schemes have to balance two factors in setting contributions: the average risk of claiming and income-based preferences. In a flat-rate contribution environment (i.e. no income differentiation), as incomes rise contribution rates decline as a proportion of income. Higher income groups, regardless of health status, prefer and can consequently afford more comprehensive benefits. Comprehensive options consequently attract multiple risk groups, even if there are disproportionately more poor risks.

Significant market pressure consequently exists to keep the better risk groups in the comprehensive options as they stabilise the pricing for their lucrative corporate clients who demand comprehensive coverage. Virtually all open schemes therefore deliberately over-price their low-cover options to cross-subsidise their comprehensive options.

Without risk-equalisation, however, government cannot easily expand the package of mandatory minimum benefits as this would compel schemes to consolidate options and destabilise the pricing of schemes with older and sicker demographic profiles. Scheme consolidation would also accelerate through the death spiral mentioned earlier, with a market equilibrium reached only when all the remaining schemes are broadly similar in demographic profile, which is most likely after significant consolidation. Although technically the expanded package with and without risk-equalisation at the new equilibrium point could be regarded as equivalent from an equity perspective, the market dynamics should be different. The extent of scheme consolidation likely in the without risk-equalisation scenario, while increasing the capability to manage provider costs, may reduce competition to such an extent that medical scheme contribution increases would have to be regulated.

### Cost containment

Although risk-equalisation would have incentivised schemes to manage healthcare provider costs more effectively (by forcing them to compete on price rather than risk), a degree of government involvement in the management of cost is inevitable due to the market power of private providers. Although cost increases have stabilised during the 2000s, the market power of hospitals and specialists in particular are able to apply systemic upward pressure for some time to come. Other health providers and medicines are unlikely to impact on cost as the former have limited market power and the latter are subject to increasing price regulation.

As three hospital groups dominate the private health system, schemes presently acquiesce to their price demands or face the threat of having their members balance-billed (required to pay the difference between what the scheme pays and hospital charges). As hospital coverage is the essential driver of demand for medical scheme coverage, short payments on catastrophic cover will result in members quickly switching their scheme (facilitated by open enrolment and brokers) to one willing to capitulate to the hospital groups. As schemes are prohibited from colluding amongst themselves by competition legislation, a collective stand against a threat to balance bill is not possible. Threats to cherry pick hospitals into networks are also easily repelled as the market for such contracts are small, and kept that way by the hospital groups. The hospital groups express their market power by preventing the emergence of price competition in the market for hospital services, thereby giving them near complete control over their revenue flows, constrained only by the number of specialists [16].

Although specialists are essential to the development of a selective contracting market for both their own and hospital services, were they to begin to enter in to such arrangements the hospital groups are likely to retaliate through the denial of hospital privileges. Thus although specialists have market power in relation to schemes, their power over the concentrated hospital system is weak. Hospital groups consequently compete, but not on price. They compete for specialists. This non-price competition drives costs up and not down, and constitutes a form of market failure quite unique to health markets.

A strategy that would have gone a long way toward balancing the market, recommended by the Taylor Committee of Inquiry in 2002 but never implemented, was to boost the autonomy and capability of public hospitals and to permit them to compete with private hospitals and specialists for the patronage of medical schemes. Unfortunately, over the past fifteen years public hospitals have deteriorated. The general low regard for public hospitals consequently makes them unattractive for medical scheme members and removes the leverage schemes could otherwise exercise over private hospitals.

### Managing non-price competition

Resolving price determination within the non-price competition market for hospitals and specialists would go a long way toward cost management within medical schemes. Proposals in this regard [25] involve the establishment of an independent (both of government and the private sector) price regulator to coordinate centralised negotiations between medical schemes, final consumers of healthcare, and all providers. The resulting prices and tariffs would apply exclusively to the non-price competition market. Any market participants willing to negotiate agreements on a selective contract basis (between schemes and non-colluding healthcare providers and suppliers) would be permitted to do so provided they complied with existing competition laws.

The proposed centralised negotiations would therefore balance out market power differences between funders, consumers and healthcare service providers resulting in more balanced pricing. Irresolvable disputes in the negotiations would be managed by automatic referral to an independent arbitrator who would decide which competing bid to accept. The arbitrator would not be free to make a separate proposal. The final decision by the arbitrator would be required to take account of factual motivations provided by the relevant parties to the initial negotiations. The overall process would generate price transparency and reasonableness in the bidding process. It would also systematically remove the abuse of market power as a basis for price setting. Importantly prices remain determined by way of negotiation between the principals rather than by government (i.e. an administered price) with government’s role to design a fair process for the negotiations [25].

## The relationship between regulated medical schemes and the public sector

The performance of the public sector has deteriorated considerably over the past fifteen years, for reasons unrelated to resourcing [7], although some have argued on the basis of crude per capita expenditure differentials between medical schemes and the public system are to blame [26]. However, health workforce differentials between the public and private sectors, apart from specialists, do not reflect an equity problem or a resourcing problem when the official registration data is corrected for errors [27-29]. Per capita expenditure differentials only suggest different cost-efficiencies and preferences between the two sectors and no plausible relationship to public health system outcomes is detectible.

Both the 1995 and 2002 committees of inquiry dealing with health reform took the view that the relationship between the non-contributory and contributory regimes of the health system should be harmonized, recognising that both offered strong mechanisms which together could efficiently achieve universal coverage. However, for the complete system to be in place specific reforms in both the public and private sectors are required. The former requires a better structuring of its institutions to enhance accountability and decision-making, while the latter *inter alia* requires risk-equalisation and cost management. It was furthermore proposed that the public and private sector providers need to be available for contracting by both the public sector and medical schemes. However, although medical schemes have the capability to purchase public sector services, the public sector is unable to manage complex contractual arrangements or meet requirements regarding quality.

## Conclusions

The South African health system provides an important case study for developing countries on mixed models for the expansion and deepening of coverage. Although it is an upper-middle-income country its experiences are nevertheless relevant to a much wider category of country contexts. Both the successes and failures are instructive. Whereas industrialised (high-income) countries have the option of achieving far deeper universal coverage via predominantly universal mechanisms and entitlements, developing countries need to confront the reality of multiple financing and service-delivery mechanisms. Private markets will also emerge regardless of their desirability. Explicitly designed mixed system models are able to combine publicly provided and subsidised services together with regulated insurance markets to effectively achieve multiple social and public goals, while leveraging off private incentives and preferences. However, achieving social protection objectives through private markets requires strong regulators and an understanding of market dynamics that are not universally well understood or documented.

The objective of universal coverage can be seen in two dimensions, horizontal extension and vertical deepening. Private systems play an important role in deepening coverage by mobilising revenue from income earners for health services over-and-above the horizontal extension role of public systems and related subsidies. South Africa provides an example of how this natural deepening occurs whether regulated or unregulated. It also demonstrates how poor regulation of mature private systems can severely undermine this role and diminish achievements below attainable levels of social protection.

The mature South African system has demonstrated its sensitivity to regulatory design and responds rapidly to changes both positive and negative. When measures to enhance risk pooling are introduced, coverage is expanded and becomes increasingly fair and sustainable. When removed, however, the system becomes less stable and fair as costs rise and people with poor health status are systematically excluded from cover. This susceptibility to regulation therefore presents an opportunity to policymakers to achieve social protection objectives through the strategic management of markets rather than exclusively through less responsive systems based on tax-funded direct provision.

As private markets will emerge regardless of government policy, any failure to effectively regulate, encouraged by the belief that commercialised health markets are inherently in opposition to the public interest, will prove counterproductive as cost spirals occur and coverage fails to deepen. Understanding the specific requirements for a well-regulated private health system within developing country contexts therefore needs to become better understood if the objectives of universal coverage are to be adequately met in the next two decades. This is especially relevant for those developing countries experiencing rapid economic growth where burgeoning unregulated private health systems are likely to emerge. This should however go hand in hand with the development of a consensus on the essential ingredients for a well-functioning general tax funded publicly provided or funded health service.

## Competing interests

The author declares no competing interests.

## Authors' contributions

Only one author was responsible for full manuscript.
